# Supplementary material for: Size, demography, ownership profiles, and identification rate of the owned dog population in central Italy
Source: PLoS One. 2020 Oct 15;15(10):e0240551. doi: 10.1371/journal.pone.0240551 (PMC7561154; doi:10.1371/journal.pone.0240551)
Supplement: S1 Table — (DOCX) [file pone.0240551.s001.docx]

| **Outcome** | **Exposure variables** |
| --- | --- |
| *Dog registry*  (No/Don’t know, Yes) | Sex (Male, Female)  Age (≤2, 2.1-8, >8)  Breed (Crossbreed, Pedegree)  Source (Born in house, Found, Adopted, Gift, Purchased)  Neutered (No, Yes)  Feeding (Homemade, Commercial, Mixed)  Veterinary visit (Never, 1-2 times, 3 or more times)  Habitat (Urban, Rural)  Living environment (Indoors, Outdoors)  Family Member (1, 2, ≥3)  Children (No, Yes) |
| *Veterinary visit*  (Never, 1 or more times) | Sex (Male, Female)  Age (≤2, 2.1-8, >8)  Breed (Crossbreed, Pedegree)  Source (Born in house, Found, Adopted, Gift, Purchased)  Neutered (No, Yes)  Feeding (Homemade, Commercial, Mixed)  Habitat (Urban, Rural)  Living environment (Indoors, Outdoors)  Dog Registry (No/Do not know, Microchip/Tatoo)  Family member (1, 2, ≥3)  Children (No, Yes)  Cat (No, Yes)  Other pet (No, Yes) |
|  |  |
| *Dog owners*  (No, Yes) | Gender (Male, Female)  Age (≤19, 20-29, 30-39, 40-49, 50-59, ≥60)  Marital status (Single/Separated/Widowed, Married)  Education level (Primary school, Middle school, High school, University)  Occupation (Home working, Office, Other)  Habitat (Urban area, Rural area)  Family member (1, 2, ≥3)  Children (No, Yes)  Cat (No, Yes)  Other pet (No, Yes) |
