# Supplementary material for: Size, demography, ownership profiles, and identification rate of the owned dog population in central Italy
Source: PLoS One. 2020 Oct 15;15(10):e0240551. doi: 10.1371/journal.pone.0240551 (PMC7561154; doi:10.1371/journal.pone.0240551)
Supplement: S1 Questionnaire — (PDF) [file pone.0240551.s004.pdf]

ISTITUTO ZOOPROFILATTICO SPERIMENTALE
DELLE REGIONI LAZIO E TOSCANA
Via Appia Nuova, 1411   00178 Roma / Capannelle
Tel. 06 790991 – www.izslt.it

Interview n° ________

The Istituto Zooprofilattico Sperimentale Lazio e Toscana is encharged in a project regarding pets tumours. IZSLT needs to estimate the pet population living in Roma Province. We kindly ask you few minutes to answer to some questions.
The questionnaire is completely anonymous and no personal data will be collected or stored

Place: ___________________ Date __ __ /__ __ /__ __ __ __   Interviewer: _________________________________

1) Do you accept the interview ?

☐ yes   ☐ no ⟶ thank you, have a nice day ***go to personal info and register sex and age class***

↓

2) Is RM 6 your LHU?

☐ yes   ☐ no   ☐ I don't know ⟶ can you tell us where do you live _________________

↓

3) How many and which animals do you have?

dogs - number: _______   *(if 0 dog but cat go to question 12)*

cats - number: _______

other- specify the species (bird, ferret): ________________________ number: _______

species (bird, ferret): ________________________ number: _______

4) If you have dogs, can you specify sex, breed and age?

	Sex		Breed	Age (Y for years M for month)
Dog 1	☐ M ☐ F	☐ neutered	☐ crossbreed ☐ breed (specify) __________	age _____________
Dog 2	☐ M ☐ F	☐ neutered	☐ crossbreed ☐ breed (specify) __________	age _____________
Dog 3	☐ M ☐ F	☐ neutered	☐ crossbreed ☐ breed (specify) __________	age _____________

if more use another form

5) How did you get the dog?

☐ born at home   ☐ found   ☐ adopted (kennel)   ☐ bought   ☐ a present

6) How many people live with the dog? _______

7) Are there children?   ☐ yes   ☐ no

8) Where does the dog live?

☐ urban   ☐ mainly INDOORS ⟶ floor____

☐ rural   ☐ mainly OUTDOORS (garden, balcony)

9) What kind of feeding?

☐ home made   ☐ pet food   ☐ mixed   ☐ I don't know

10) How many times does the dog go to the vet per year?

☐ never   ☐ 1-2   ☐ 3 or more

11) Is the dog registed in the Dog Registry?

Dog 1	☐ YES tattoo	☐ YES microchip	☐ YES I don't know	☐ NO	☐ I don't know
Dog 2	☐ YES tattoo	☐ YESmicrochip	☐ YES I don't know	☐ NO	☐ I don't know
Dog 3	☐ YES tattoo	☐ YES microchip	☐ YES I don't know	☐ NO	☐ I don't know

12) If you have cats, can you specify sex, breed and age?

	Sex			Breed		Age (Y for years M for month)
Cat 1	☐ M	☐ F	☐ neutered	☐ crossbreed	☐breed (specify) __________	age _____________
Cat 2	☐ M	☐ F	☐ neutered	☐ crossbreed	☐breed (specify) __________	age _____________
Cat 3	☐ M	☐ F	☐ neutered	☐ crossbreed	☐breed (specify) __________	age _____________

if more use another form

13) How did you get the cat?

☐born at home ☐ found ☐ adopted (kennel) ☐ bought ☐ a present

14) How many people live with the cat ? ______

15) Are there children? ☐yes ☐ no

16) Where does the cat live?

☐urban ☐ mainly INDOORS ⟶ floor____

☐rural ☐ mainly OUTDOORS (garden, balcony)

If you are in doubth: (no four-sised houses)

17) What kind of feeding?

☐ home made ☐ pet food ☐ mixed ☐ I don't know

18) How many times does the cat go to the vet per year?

☐ never ☐ 1-2 ☐ 3 or more

19) Is cat registed in the Feline Registry?

Cat 1	☐ YES microchip	☐ YES I don't know	☐ NO	☐ I don't know
Cat 2	☐ YESmicrochip	☐ YES I don't know	☐ NO	☐ I don't know
Cat 3	☐ YES microchip	☐ YES I don't know	☐ NO	☐ I don't know

Anonymus personal information

Sex ☐ male ☐female

Age ☐ 0-19 ☐ 20-29 ☐ 30-39 ☐40-49 ☐50-59 ☐ >= di 60

Marital status ☐ single ☐married /long term ☐ divorced/separated ☐ widowed

Educational qualification ☐ primary ☐ secondary ☐ high school ☐ degree

Job

☐ student ☐ unemployed ☐housewife ☐jubilee ☐other : ________

☐ craftsman ☐ office worker ☐workman ☐ freelance
