# Supplementary material for: Size, demography, ownership profiles, and identification rate of the owned dog population in central Italy
Source: PLoS One. 2020 Oct 15;15(10):e0240551. doi: 10.1371/journal.pone.0240551 (PMC7561154; doi:10.1371/journal.pone.0240551)
Supplement: S2 Questionnaire — (PDF) [file pone.0240551.s005.pdf]

ISTITUTO ZOOPROFILATTICO SPERIMENTALE
DELLE REGIONI LAZIO E TOSCANA
Via Appia Nuova, 1411 00178 Roma / Capannelle
Tel. 06 790991 (centralino) – Fax 06 79340724

Intervista n° **nnn**

L'Istituto Zooprofilattico Sperimentale Lazio e Toscana è attualmente impegnato in un progetto per lo studio dei tumori in cani e gatti. Per il successo di questa iniziativa è necessario stimare la popolazione dei cani che vivono all'interno della ASL RM H. Le chiediamo gentilmente pochi minuti per rispondere a poche domande. Il questionario è anonimo e in nessuna occasione le veranno chiesti i suoi dati personali

Luogo: ________________ Data ____/____/_____ Intervistatore: ____________________________________

1) Accetta l'intervista ?

☐ si ☐ no → grazie lo stesso, buona giornata

↓

2) La sua ASL è la RM H ?

☐ si ☐ no ☐ non so → vuole dirci il comune di residenza (cani) ________________

residenza o domicilio (gatti) ________________

↓

3) quali e quanti animali vivono con lei (o comunque sono sotto la sua tutela) ?

cani - specificare il numero: ______

gatti - specificare il numero: ______

altro - specificare la specie (uccelli, furetto): ______________________ e il numero: ______

specie (uccelli, furetto): ______________________ e il numero: ______

se 0 cani ma ha gatti vai DIRETTAMENTE alla domanda 10

4) se con lei vivono dei cani, mi può dire il loro sesso, razza ed età ?

	Sesso			Razza		Età (A per anni M per mesi)
Cane 1	☐ M	☐ F	☐ steril	☐ meticcio	☐ razza (specificare) _________	età ____________
Cane 2	☐ M	☐ F	☐ steril	☐ meticcio	☐ razza (specificare) _________	età ____________
Cane 3	☐ M	☐ F	☐ steril	☐ meticcio	☐ razza (specificare) _________	età ____________

5) di quante persone si compone la famiglia che vive con il cane ? ______

6) ci sono bambini ? ☐ si ☐ no

7) in che tipo di ambiente vive il cane ?

☐ centro urbano
- ☐ prevalentemente al chiuso → a che piano____
- ☐ prevalentemente all'aperto

☐ campagna
- ☐ prevalentemente al chiuso → a che piano____
- ☐ prevalentemente all'aperto

definizione di campagna: la casa dove vive l'intervistato non ha altre case in nessuno dei 4 lati

8) che tipo di alimentazione ha il cane ?

☐ casalinga ☐ industriale ☐ mista ☐on so

9) il cane/i che vivono con lei sono iscritti all'anagrafe ?

Cane 1	☐ SI tatuaggio	☐ SI microchip	☐ SI non so	☐ NO	☐ non so
Cane 2	☐ SI tatuaggio	☐ SI microchip	☐ SI non so	☐ NO	☐ non so
Cane 3	☐ SI tatuaggio	☐ SI microchip	☐ SI non so	☐ NO	☐ non so

10) se con lei vivono dei gatti mi può dire il loro sesso, razza ed età ?

	Sesso			Razza		Età
Gatto 1	☐ M	☐ F	☐ steril	☐ meticcio	☐ razza (specificare) _________	età _____________
Gatto 2	☐ M	☐ F	☐ steril	☐ meticcio	☐ razza (specificare) _________	età _____________
Gatto 3	☐ M	☐ F	☐ steril	☐ meticcio	☐ razza (specificare) _________	età _____________

11) di quante persone si compone la famiglia che vive con il gatto ? ______

12) ci sono bambini ? ☐ si ☐ no

13) in che tipo di ambiente vive il gatto ?

☐centro urbano
- ☐ prevalentemente al chiuso
- ☐ prevalentemente all'aperto

☐ campagna
- ☐ prevalentemente al chiuso
- ☐ prevalentemente all'aperto

14) che tipo di alimentazione ha il gatto ?

☐ casalinga ☐ industriale ☐ mista ☐non so

15) il gatto/i che vive con lei è/sono iscritti all'anagrafe ?

Gatto 1	☐ SI tatuaggio	☐ SI microchip	☐ SI non so	☐ NO	☐ non so
Gatto 2	☐ SI tatuaggio	☐ SI microchip	☐ SI non so	☐ NO	☐ non so
Gatto 3	☐ SI tatuaggio	☐ SI microchip	☐ SI non so	☐ NO	☐ non so

La ringraziamo per sua la disponibilità e il tempo che ci ha dedicato

Note

____

____

____
