# Supplementary material for: Size, demography, ownership profiles, and identification rate of the owned dog population in central Italy
Source: PLoS One. 2020 Oct 15;15(10):e0240551. doi: 10.1371/journal.pone.0240551 (PMC7561154; doi:10.1371/journal.pone.0240551)
Supplement: Attachment — Submitted filename: Response to Reviewers.docx [file pone.0240551.s006.docx]

Dear Editor,

we are pleased to submit the revised manuscript: “Size, demography, ownership profiles and identification rate of the owned dog population in central Italy”.

We thank the Editor and both reviewers for their comments, which greatly improve the manuscript.

We have followed your instructions in revising the manuscript and put the comments in tables to facilitate the revision process. One comment regarding the subsections of a reviewer is in conflict with revisions requested by the other one. We apologize but we can not accept both requests.

We also add in the manuscript and in the revision process in PlosONE website, the data availability and the details on interviews as requested by a reviewer.

We hope that, after the performed changes, the article meets the target and the requirements of Your Journal.

Sincerely

Andrea Carvelli

---------------------------------------------------------------------------------------------------------------------

Reviewer #1

|  | Regarding availability of all data. The authors state that all data is available in the manuscript itself. I think that the journal is looking for raw data. | Reviewer is right. We will upload the overall excel database in Supporting Information.  We will upload the questionnaire also, both in original language and in English. |
| --- | --- | --- |
|  | This manuscript attempts to better understand and clarify some important information about the dog population in the region around Rome. Part of the challenge is that there are at least 4 objectives: 1. estimate the owned dog population size and demographics; 2. Determine the proportion of dogs not registered with the national registry and see if there are variable which predict this; 3. Predictors of annual veterinary care; 4. Predictors of dog ownership. And the authors spend a lot more time on #2 than the others in both the intro and discussion. The justification and recommendations are rather superficial and simplistic even though the authors are clearly passionate about this topic. | Reviewer is right. We edit the manuscript adding text regarding the other objectives of the paper, demography, veterinary care predictors and ownership predictors. |
|  | The manuscript is hampered by the need for English language editing. It is quite wordy and repetitive regardless. I would suggest that the authors organize the manuscript according to the 4 objectives, particularly in the methods, results and discussion. | A mother tongue consultant performed a thorough review for English.  We introduced subsections also in the Introduction and we leave the subsection in the Discussion.  It is authors’ opinion that dividing into subsection the whole manuscript can lead to redundancies. For example, the sentence at the beginning of the Results section “*The interviewed people was 630 (omissis) and 455 people were considered in the analyses (omissis). The total number of interviewed people and their family members was 1,297. (omissis) Overall, 244 (53%) interviewed people did not have dogs while 212 (47%) owned at least one dog. Of these, 140 (66%) owned one dog, 53 (25%) two dogs, 13 (6%) three dogs, 6 (3%) four or more dogs, counting up to 315 dogs*” is useful for 3 out of 4 objective. Moreover, M&M is already divided in subsections that we would like to leave – study area, questionnaire, data analysis.  Moreover, the other review asked to remove the subsections also from the discussion… |
|  | The introduction is focused on identification and registration, an important issue for the reasons outlined. How then does the paragraph beginning on line 74 fit? The other objectives? | We moved the sentence L74.  We edit the manuscript adding text regarding the other objectives of the paper, demography, veterinary care predictors and ownership predictors. |
| L 63 65 | in the former line the authors state that identification and registration are mandatory and in the latter that registration isn’t required. I think this may be a language issue but I’m not sure | The sentence was rephrased to be more clear as follows: “*In most European countries, dog identification and registration (I&R) have become mandatory in recent years [7]. Nevertheless, while identification is performed on individual basis, registration in a central accessible database is not compulsory in some country or not implemented effectively in others*.” |
| L 67 | Is there a relevant reference for the 2016 earthquake data? | Unfortunately, there is no reference but only personal experience and power point presentation in local meetings. |
|  | And if there are already data about registration rates in Rome, what does this study add to that information? That should be in the introduction as well. | The registration rate has been already investigated but in a different area of Rome and some years before.  We modified the sentence as follows: “*A previous study carried out to assess the completeness of the DR in a high densely populated area of Rome found that only the 75% of the dogs were registered [9]. These findings show that the real owned population is likely underestimated in DRs. Therefore, a periodic assessment of the DR completeness is appropriate and factors influencing owners to register a dog are worth to be investigating*”. |
|  | If this manuscript is also about dog population demographics, how does that relate to the dog registry work and the 3rd and 4th objectives? That should succinctly be included in the introduction. | Reviewer is right. We edit the manuscript adding text regarding the other objectives of the paper, demography, veterinary care predictors and ownership predictors. |
| L 108 | Please include a few sentences briefly describing the study area so the reader doesn’t have to find and read another paper to learn this | We modify the sentence and added details. |
| L 109-14 | I don’t understand what was done here. I realize the authors were trying to estimate sample size, but it seems to be for two different pieces of work (yet this is one study) and two different confidence intervals. Were the authors looking for a sample size to provide a specific level of precision on the estimates? And since the two sample sizes are quite different which one was used? Only the larger one is needed if that was used. | Sample sizes were different for the different objectives of the paper.  The assumptions needed for the evaluation of the DR (question to be answered: how many dogs are correctly registered?) (expected prevalence: 75%; standard error: 5%; confidence level: 90%) led to a sample size of 203. This sample size had to be applied and referred to people owning at least one dog.  The assumptions needed for the study of demography and ownership profile (question: how many people own a dog?) (expected prevalence: unknown; standard error: 5%; confidence level: 99%) led to a sample size of 664. This sample size had to be applied and referred to people owning or not a dog.  So, both sample sizes were needed. We had to interview at least 664 people. And we had to interview at least 203 people owners of a dog.  As the assumptions and the objectives are different, it is authors’ opinion that it is important to specify both calculation for sample size. We hope that after this explanation, the reviewer agrees. |
| L 119 | This is a convenience sample which the authors argue is “representative” in the companion cat paper. A few sentences explaining how the respondents were selected is needed here. And in the discussion some justification of why this would be considered representative. | We added in M&M the following sentences: “*The survey was carried out during weekdays between the months of July and December 2013 in the waiting rooms of 4 General Health Care Centres of the National Health System. A systematic sampling was performed by selecting one every four person amongst the general diagnostic patients. Survey participation was requested within the framework of a research project on human and animal tumours managed by the Ministry of Health*”  We added in Discussion the following sentences: “*The approach used in the present paper, a face-to-face questionnaire, thought costly and time-consuming, improves the precision of the estimates limiting the selection, non-response and the measurement biases]. The questionnaires were asked in Health Care Centres to general people attending for booking, payment or routine diagnostic exams (blood analyses, general medical visits, clinical examinations). In Italy, the Health Care System provides comprehensive coverage to all citizens. Considering that routine diagnostic exams are performed in the Health Care Centres and homogenously among age, sex and socio-economic levels, the sampled population can be considered representative of the general population.”* |
| L 151 | excluded dogs > 16 years old but only present data in figure 1 at 13 years and up. Please be consistent and give some explanation for the choices. | We consider dead the dogs > 16 years old because we assume that after 16 a dog should be dead. In a study authors found that after 16, there is a consistent drop of the survival rate (https://doi.org/10.1111/acel.12737). So, we assume that if a dog is still present in the DR, the probability that the dog is still alive is minor than the dog is dead and not removed from the DR.  In the population pyramid, we use to put the dog that are 14, 15 and 16 years old in the same class because of their small number. |
| L 152 | so, all pairwise combinations of the dog characteristics were tested against each other? Why? And what if the expected frequencies were < 5? At least state how many dog characteristics there were and therefore how many tests were done. And what about multiple comparisons with a p-value of 0.05 overall? | We applied chi square test while including each variable in the multivariable model to avoid multicollinearity. The variables significantly associated with the outcome were tested about all pairwise combinations before being entered in the multivariable model.  Besides chi square test, we applied Fisher exact test for expected frequencies <5.  We added it in the text as follows: “*The risk factors with a bivariate p value ≤0.25 were included in a multivariable stepwise logistic model. Chi square or Fisher exact test were used to evaluate a possible multicollinearity among risk factors that were significantly associated with the outcomes.*” |
| L 153 | What exposure variables were used? I’d like to see a list but a reference to a table or appendix would be ok. State how many there were. And that the other two dependent variables were used as independent variables in the other models. | We added a Supplementary Table with the outcome and the exposure variables considered in each analysis/model. |
| L 156 | The likelihood ratio test tells you which model fits better in comparing models so that you can decide which variables to include. It doesn’t test whether the final model meets the assumptions for logistic regression. That still needs to be evaluated and included here and in the results. | We performed the likelihood ratio test to compare the last model with full models (in which appeared all the significant variables in the univariate analysis. We modified the text as follows: “*The likelihood ratio chi square test (LRT) for goodness of fit was calculated to compare the final model with full models.*”  The variables included in the model were categorical and not pairwise associated, therefore the assumptions of the final model were fulfilled. |
| Results | Were all continuous data normally distributed so that the mean is a valid measure of the central tendency? Please add to the methods and adjust to a median if needed | All the considered variables were categorical, so in M&M we stated that absolute frequency and percentage were used.  The only mean and SD used was for the dog age but no statistical analyses were made on it. When statistical analyses on age were done, the variable was categorized. |
| L 172 | I understand using a Bayesian approach for the estimate but if only 58% of dogs were registered in the sample, then how can the overall estimate be so much higher? Seems like there is something missing here. | The value mentioned by the Reviewer (58%) represents the observed proportion of dogs that live in Lazio Region and have been registered by the owners. We took in account this difference/gap between the actual regional dog population and the population size based on the registry data applying the formula reported in M&M section. The adopted underestimation factor was calculated using a Bayesian approach and derives from data provided by a similar previous investigation. |
| L 179 | Were these the only dog characteristics that were significantly associated? Please provide actual p-values. | Reviewer is right, it was unclear. The variables were tested as already explained in comment L152 and in the revised paper. Line 179 was another comparison but it was useless because the difference is so big that we do not need statistics to tell us that there is a difference. Therefore, we modified the sentence removing the p. |
| Table 1 | given that the subsequent tables have descriptive data for each logistic model this table isn’t needed. | Reviewer is right, the data of table 1 are present also in the logistic analyses tables, but the way to present it in a simple and direct way (as in Table 1) could be useful to readers that are interested in demography. They could not find easy to recalculate the characteristics biased by the outcome variable in subsequent tables. |
| Tables 2-4 | please add the actual univariable p-value to the variable row. Then the superscript * will not be needed. | We choose superscript * for reading easiness. Adding every P values would mean add other two columns probably making tables more difficult to read and too large for journal standards. If reviewer agree, we think leaving the p value as legend makes tables clear. |
|  | You could additionally bold the variables that stayed in the multivariable analysis.  Were any interactions considered? There was an association between sex and neuter status, was that examined or a new variable created and used as was shown in Table 1?  Why weren’t the owner demographics included in the registry and veterinary care models? | We bold the variables that are significantly associated with the outcome in the multivariable models.  See previous comment (L 152)  The ratio between dog and owner is not 1:1. Many owners have more than one dog. The dataset used for Table 2 and 3 is different from the subdataset used for Table 4. If we added owners’ demographics, owners with more than one dog would be overrepresented. |
|  | The first part of the discussion feels very generic and not really related to this manuscript and study. Please focus on the objectives of the study and how they relate to important other studies and specifically how they can be used in the future. Order the discussion based on the objectives and don’t include a lot of repetition of results or every article and comparison. This should be a thoughtful and critical discussion of what the results add to the knowledge base, what the limitations are and what can be done in the future. I have only included a few comments below. | We edit the manuscript following Your suggestions. |
| L 234 | and following seems like it is related to the limitations of this study? That should be its own section later in the discussion with all other pertinent issues. | Actually, it is an introduction sentence that tried to explain why this study has a low selection, non-response and measurement bias (L 237).  In the revised manuscript we added a part on limits of the study in the Discussion section. |
| L 253 | the term poorer human development index is used several times in the manuscript without definition. And the references here are for countries that are considered to be developed except for references 23 and 24 which might be considered to be poorer or developing. Please edit. | We put the references of the whole sentence all together at the end to avoid reading problems. If the reviewer think it is better, we could modify the sentence as follows: “*The percentage of respondent having a dog was 47%, much higher than previous findings in Italy (33%, 25%)[17,28]and in Europe (39%, 31%)[14,16], but lower if compared to countries with a poor Human Development Index (65%, 51%) [29,30].”* |
| L 279 | reference 26 is a tumor registry not a dog identification and registration paper. | Reviewer is right. It is a paper on tumours. Actually, as authors calculate tumours incidence, they first calculate the denominator, by dogs identification and registration. |
| L 292-3 | the authors have a very traditional view that if only the owners were more “responsible” things would be better. That is a very judgmental and often inaccurate and unhelpful portrayal of pet owners. There are real barriers in some cases for owners to do what they should. In fact, alternative reasons to those stated in this sentence could be that they didn’t know they were supposed to register the dog, didn’t know how or what to do, hadn’t had time yet to do it, etc. Please edit. | The responsible ownership is a fundamental tool in dog population management that is presented by Institutions (European Union, OIE, FAO), Veterinarians (ANMVA, FVE) and charities. We added a FAO and OIE reference and related definition by OIE in the Introduction.  Moreover, we added in the introduction section some sentences trying to explain why in Italy we are confident that most of the population is aware of the duty of dog I&R: “*Since the DR establishment, many information campaigns have been organised toward general population through radio, newspaper, social media, leaflet and advertising posters. The identification and registration costs are affordable*. |

Reviewer #2:

| Page 4, line 69 | “A number of papers have been published on owned dog population estimate”. I think this sentence seems lost and it can be deleted. | We moved the sentence L74 that is now a sort of introduction to dog population abundance argument. We hope that the new order makes sense. |
| --- | --- | --- |
| Page 5, line 79 | “electronic transponder”. Is this a microchip? | We modified the sentence as follows: “*In Italy, since 1991, every owned dog and stray dogs caught by the competent authorities must be identified by an electronic transponder (microchip) and registered in a DR (Framework Law 281/1991)*.” |
| Page 5, lines 84-85 | Please briefly explain the main purpose of the DR. | We modified the sentence as follows*: “Most of the people think that the only purpose of DR is the reunification of lost dogs with their owners, underestimating the epidemiological relevance of population abundance assessment”.* We also added a sentence on the main purpose of the Law that established the DR. |
| Page 6 | Questionnaire design section: How the individuals were selected?  Did the authors interview the head of the household or any available individual?  I also missed the ethic consideration regarding the interviews. | Reviewer is right, we added in M&M the following sentences: “*The survey was carried out during weekdays between the months of July and December 2013 in the waiting rooms of 4 General Health Care Centres of the National Health System. A systematic sampling was performed by selecting one every four person amongst the general diagnostic patients. Survey participation was requested within the framework of a research project on human and animal tumours managed by the Ministry of Health*”  We added in Discussion the following sentences: “*The approach used in the present paper, a face-to-face questionnaire, though costly and time-consuming, improves the precision of the estimates limiting the selection, non-response and the measurement biases [2]. The questionnaires were asked in Health Care Centres to general people attending for booking, payment or routine diagnostic exams (blood analyses, general medical visits, clinical examinations). In Italy, the Health Care System provides comprehensive coverage to all citizens. Considering that routine diagnostic exams are performed in the Health Care Centres and homogenously among age, sex and socio-economic levels, the sampled population can be considered representative of the general population [21]”.*  We interviewed only one person of the household. Indeed, in the Result section we wrote: “*A total of 630 people were interviewed, the answer rate was 76% and 455 people were considered in the analyses because they resided in the study area. The total number of interviewed people and their family members was 1,297.*”  The present study involves human participants only for interviews. The risk for harm the environment or the person was null. No practice on human or animal nor intervention was made. The data collected during interview were completely anonymous, thus no form of consent was asked. Collected data only contain information regarding owned dog and age, marital status, education and occupation of the interviewed person. Before starting the interviews, people were verbally asked to partecipate and if they refused, no data was registered. Individuals enrolled in the interviews freely agree to do it. The interviews were performed in a waiting room, a place where every people was free to enter or to leave.  Before starting the activities, some e-mail and phone calls were made between Dr Scaramozzino, the Head of Epidemiology Unit of Istituto Zooprofilattico Sperimentale and Dr Sigismondi, Head of Dipartimento di Prevenzione of Local Health Unit. Dr Sigismondi agreed to allow interviews and no Ethics Committee was asked to give permission. We specified it in the submission process in PLoS ONE website. |
| Page 8, lines 161-164 | These are interesting results regarding the human population. However, a demographic description of your human population is missing, and it would add more value to your manuscript. Also, I think authors should provide an additional table for the human demographics. | Reviewer is right, it could be valuable. Actually, there are some limits regarding this option. First, we have some missing on demographic information on people not owning dog that didn’t give the permission to collect data on them.  Secondly, the ratio between dog and owner is not 1:1. Many owners have more than one dog. The dataset used for Table 2 and 3 is different from the subdataset used for Table 4. If we added owners’ demographics, owners with more than one dog would be overrepresented.  Finally, the paper is quite long. Table 4 is already for human demographics, also if only referring to dog owners. If reviewer agree, we would not like to add another table. |
| Page 16, Discussion | I would not write the discussion in topics. Its preferable to be a flowing text as in the background. | Reviewer could be right, but the other reviewer asked us to add topics also in Introduction, M&M, Results and to leave them in Discussion! It is very subjective. We will ask the Editor which reviewer suggestion to consider. |
|  | The main conclusion and limitations of the study are missing. | We edit the manuscript following Your suggestions. |
