# Supplementary material for: Size, demography, ownership profiles, and identification rate of the owned dog population in central Italy
Source: PLoS One. 2020 Oct 15;15(10):e0240551. doi: 10.1371/journal.pone.0240551 (PMC7561154; doi:10.1371/journal.pone.0240551)
Supplement: Attachment — Submitted filename: Response to Reviewers R2.docx [file pone.0240551.s007.docx]

Dear Editor,

We are pleased to submit the revised manuscript “Size, demography, ownership profiles and identification rate of the owned dog population in central Italy”.

We thank the Editor and both the reviewers for their comments, which greatly improve the manuscript.

We have followed your instructions in revising the manuscript and put comments in tables to facilitate the revision process.

We hope that, after the performed changes, the article meets the target and the requirements of Your Journal.

Sincerely

The Corresponding Author

---------------------------------------------------------------------------------------------------------------------

Reviewer #1

|  | The manuscript is understandable with the revisions including the English language edits, but still in need of some editing for English as well as to reduce repetition and make the manuscript more concise, clear and focused. I have therefore not commented line by line, only where I noted obvious needs for editing. Having someone who has written many scientific articles in English language journals review and edit the manuscript would be very helpful! | The manuscript has been revised according to Your comment. A mother-tongue consultant performed a thorough review for English.  Specific revisions are presented in following points. |
| --- | --- | --- |
|  | The Introduction is still too long and a bit repetitive. It moves from national data to Italian registration to international need for data to ownership data in other countries. Please review some recent Plos One articles to see examples of the construction of the introduction. The previous cat focused paper is also an example where the introduction was more focused but don’t use the same sentences--any exact repetition is plagiarism. See as an example Mustiana 2015 in Plos One. Order the topics from most general (international) to most specific (Italy/Rome). | In revision#1 authors were asked to add some comments on the 4 objectives of the paper. This made the introduction longer.  Each objective is introduced separately (in different paragraph) as requested in revision#1, and follows the general rule, ‘from general to specific’ in each paragraph. We changed the order of some paragraph and removed some redundancies according to Your comment. |
| Line 88 | Note that any fee even a small one, for someone who is very poor may be cost prohibitive. This is an assumption that is untested. | Revised. We added the cost. |
| Line 140-2 | To be able to say that selection bias was limited, you need to state what was done if the selected person refused to participate here and, in the results, indicate how many people refused to participate. | When a person refused to participate, interviewers select the following person, with the same criterion, always with a pace of 4.  Some people refused to participate, the response rate was already declared at the beginning of the Results section. |
| Page 8 | Age and number of pets were collected as continuous variables. How did the authors determine what categories to create? Based on what?  Gender is applied only to humans. In animals, sex is the preferred term. | Age classes were arbitrarily chosen based on young/adult/old dogs categories, according to a biological criterion, commonly used in scientific literature.  We used ‘sex’ for pet and ‘gender’ for humans. Also if another reviewer asked us the opposite. |
| Lines 167-8 | These sentences are not clear. I think, based on the companion cat study, that the authors are indicating that the interviewers framed the present research as a study of general pet health to avoid bias. And that whether the pet was allowed to roam was not asked? | Interviewers framed the research as a study on tumours (as it was) as stated in M&M and in discussion sections to minimise biases. See also another comment on this (L271-277). As stated in M&M section, the physical restriction of the animal was not taken into consideration. |
| Line 196 | Relative to the two sample size calculations, the authors apparently only used the sample size which they first reached, the dog ownership one. Why not also the number interviewed? That needs to be in the manuscript. | Two different sample sizes were calculated for different aims of the research. We added a sentence in M&M to be clearer. One sample size was calculated (with some assumptions) for the evaluation of the DR=203 (questionnaires to be administered to people owning at least one dog). A number of 212 was reached.  Another sample size was calculated (with different assumptions respect to the first one) for the study of the dog demography and ownership profiles=664. A number of 630 was reached.  All numbers are present in the M&M and in the Results sections. |
| Line 209 | Were the ages normally distributed? Fig 1 would suggest they are NOT normally distributed and therefore the median would be a more correct option. | Revised. We changed means and SD in medians and quartiles. |
|  | Table 1 does not appear to add information to that of Table 2. I would either delete or put into the supplement. Alternatively, one column of totals could be added to table 2. | Data of Table 1 are present also in all the logistic analyses tables, not only in Table 2, but the way to present it in a simple and direct way (as Table 1 does) could be useful to readers that are interested in demography. They could not find easy to recalculate the characteristics biased by the outcome variables in subsequent tables.  Adding totals in Table 2, 3, and 4, would be redundant and it would make tables too complicated, with |
|  | Table 2 only has 312 dogs. What happened to the other 3? | It is a missing value on the outcome variable. The same happened in Table 3. The absolute frequency of dogs are already reported in the columns headings. |
|  | Authors response to L152: does not address my question about multiple comparisons for the chi-square tests for multicollinearity. And the first sentence added is about the chi-square test between outcome and independent variables, not about multicollinearity. Were any variables excluded from any model due to statistical associations with each other? Appears they were not based on a later comment. That needs to be in the results section. | We did not perform chi square among exposure and outcome variables. We performed chi square test among exposure variables in revision#1. We now perform also phi coefficient when possible (2X2) and it resulted not significative. No statistical association was found among all independent variables included in the final models.  Interactions among exposure variables was tested in this revision.  We modified the text as follows:   - M&M “*Chi square test and phi coefficient were used to evaluate a possible multicollinearity among risk factors that were significantly associated with the outcomes. The absence of plausible interactions among variables were tested in all regression models.*” - Results “*There was no multicollinearity between the independent variables. In the regression models, interaction terms were not significant.”* |
|  | Authors response to L156: in fact, dogs living in the same household are likely not independent which is one of the assumptions for Logistic regression. Please consult with someone who can assist with the statistical analysis. | We re-run models with HH as exposure variable and it was not significative.  We added the following sentences in M&M: “*. In logistic regression analyses with DR and veterinary care considered as outcome variables (Tables 2 and 3), the HH was included in order to ensure the independence of observations (i.e. an owner owning more than one dog having the same behaviour or attitude with all the dogs). In the logistic regression analysis regarding dog ownership profiles (Table 4), the outcome variable was represented by the owner and not by the dog, avoiding the overrepresentation of HHs with more than one dog.*”  In the Results section, two sentences were added while commenting Tables 2 and 3: “*HH was found to have no effect on the outcome variable (p=0.07”. “HH was found to have no effect on the outcome variable (p=0.432)*”. |
|  | Authors response to Tables: I’m asking about statistical interactions in the final multivariable models, not multicollinearity. Please add these to the analysis.  And because the dogs are clustered or matched within households, households may need to be added to the analysis. That would also allow owner characteristics to be included. There are statistical methods to address this. And if there is a compelling reason to ignore the clustering within households, the authors must include that. | We tested for interaction and modified the paper. See previous comments on this.  Some HHs have more than one dog.  In Tables 2 and 3 we added HH as exposure variables. It did not have effect. We modified the text. Please see the previous comment on this.  In Table 4, we took in consideration only the owner and not dogs. There is no risk that HH with more than one dog was overrepresented. We added a sentence in M&M section. |
|  | I could make the argument that since the person who answered the survey wasn’t selected as someone who made the decisions for household about the dogs that their characteristics would be unlikely to be related to I&R. That should be included in the discussion as another limitation and may explain why a few HH level variables were significant, but the individual ones were not. | We added this limitation in the discussion. Thank you for raising this important point. |
|  | Table 3: please add something to the title about this including the results of 203 owners who were surveyed in x years. And add the 312 dogs to the existing description. Add a bit more to the title, similarly, for Tables 2 and 4. | Revised. |
| Lines 271-7 | Please do not repeat verbatim the section from the cat paper. And there were a number of people selected who refused to participate, no? Please include that in the results. Then adjust this statement and add to the limitations section. This influences non-response and selection biases. Measurement bias wasn’t addressed by the source of participants, only by 1. The training of the interviewers and data entry staff, 2. The validity and reliability of the survey, 3. The way the survey was presented to the respondents, etc. Please be much more careful in the discussion about this and other types of bias and how they were minimized (and if not, how they were likely to influence the results). | We modified the sentences, avoiding repeating verbatim.  Some people refused to participate, the response rate was already declared at the beginning of the Results section.  We are here stating that the method we used, face to face questionnaire, limit also the measurement bias because, as You say, we could control the training of interviewers and the way the survey was presented to the respondents. We are highlighting that this method is to be preferred because it is the one that minimize biases. We added a sentence: “*Biases were also minimised recruiting respondents within the framework of a research project on human and animal tumours and not directly on dog ownership*“. |
| Line 292 | Human development index appears in the abstract without any definition or description. And isn’t described here either only referenced. Please briefly indicate what indices are included. The next sentence mentions other indices, but none are discussion, unless the dogs per/km2 is the next index—in which case it is about indices to report dog data not potential predictors? Please clarify. | We added the explanation of Human Development Index. The following sentence is a brief part of ‘results’ to introduce the discussion about it.  We do not want to say that these indices are not potential predictors. We want to say that we found a large number of dogs in an area with an remarkable density of humans. But this can not be assumed as a potential predictor. |
|  | What is the major point of the paragraph starting on line 289? The paragraph ranges through human development index, density in Rome and other countries, and dog and cat ownership. | The major point is the abundance of dogs. Then, this point is discussed in relation to the high human density of Rome metropolitan area. |
| Line 324 | I’m not clear how the homogeneity of the I&R in age (which is quite interesting!) is related to the need for action? | We would expect young classes more identified and registered than older classes. This would have led to a more complete DR in the future as older dogs die and younger dogs become adult already identified and registered. We modified the sentence trying to be clearer. |
| Line 331 | Isn’t responsible pet ownership promoted everywhere already? Has it been shown to have an impact on I&R? If there aren’t data to support these strategies, then the authors should at least say how they think these will promote I&R and who and how they should be performed. | Responsible dog ownership improves I&R. One of the first assumption of the I&R policy is to identify and register a pet, so the pet can be referred to a person. Then, it sounds implicit. The cited reference explain the ‘Responsible dog ownership’ policy that should be known by readers of the paper. Questions raised by the reviewer are interesting, but we think that it is not the purpose of the paper to answer them, also considering that the paper is already long and complex. |
| Line 343-4 | These references are only in a handful of countries and don’t include the US or Canada. Please rephrase. | Revised. We remove worldwide. |
| Line 341 | Is this about males or neutering in general? The paragraph includes both sexes, but the previous sentences focus on males (I believe). | The first paragraph is only about sex. The second one is about neutering of both sexes. We added ‘overall’ |
| Line 362 | I believe that the authors are saying that if puppies are being imported illegally then they are likely not going to be I&R? | Revised following Your suggestion. |
| Line 366-389 | Paragraph starting on line 366 tile 389: There is good information here, but it is long and repetitive. Please edit to be shorter. | This is a new part added during revision#1, because reviewers asked to discuss more the veterinary care. However, we modified this part, trying to avoid some redundancies. |
| Line 406-407 | “also more likely to own a cat” than what? This sentence is incomplete. | Revised. The sentence was modified as follows: “Interestingly, dog owners were also more likely to own a cat than people not owning a dog”. |
| Lines 409-11 | These sentences seem to be saying that: this study was unable to find predictors to confidently predict dog ownership in this or other parts of Italy. | Reviewer is right. We wanted to say this. Few variables were statistically associated with dog ownership. |

Reviewer #3

|  | This is a nice paper, which has some interesting results from Italy. The science itself seems generally sound, and will be of interest to readers. However, it needs a bit of grammatical reworking, and I have tried to do this below as I am guessing that their first language isn’t English. The changes below don’t need a response writing to them, but hope they help. | Thank you for the revision. A mother-tongue consultant performed a thorough review for English. We edited the paper accordingly. |
| --- | --- | --- |
| Line 19 | predictors in the animal population is essential (add in word) | Revised. |
| Line 38 | The percentage of neutered dogs were higher in females (55%) than in males (8%). (reword) | Revised. |
| ine 43/44 | including promoting responsible dog ownership, (reword) | Revised. |
| Line 57 | change human to humans | Revised. |
| Line 58 | Therefore, the knowledge of the animal population in terms of size (add in word) | Revised. |
| Line 62 | the lack of information in the pet population is a constant (add in word) | Revised. |
| Line 66 | owned dog population estimates (make plural, and add in references) | Revised. The references are in the following lines of the same paragraph. |
| Line 73 | please define DRs | Revised. Done in Line 78 |
| Line 75 | Nevertheless, while identification is performed on an individual basis, (add in word) | Revised. |
| Line 76 | change country to countries | Revised. |
| Line 78 | To date, reliable data on the dog population (add in word) | Revised. |
| Line 84 | or manage data of both the (add in word) | Revised. |
| Line 86 | organised toward the general population (add in word) | Revised. |
| Line 88 | can you please put in the cost of this registration? It may be prohibitive to poor people not to register | Revised. |
| Line 90 | stakeholders think that the only purpose of the DR is the (add in word) | Revised. |
| Line 94 | remove high | Revised. |
| Line 95 | remove the | Revised. |
| Line 105 | remove an | Revised. |
| Line 106 | its consequences, such as human and animal injuries (make plural and add in comma) | Revised. |
| Line 109 | useful for the private sector (add in word) | Revised. |
| Line 112 | add in comma after evaluated | Revised. |
| Line 125 | owned dog population, and to identify the human factors…. (change final comma to and) | Revised. |
| Line 140 | what happened if a person refused to participate? Also, if this was done in 2013- how it is linked to what is relevant now? | When a person refused to participate, interviewers select the following person, always with a pace of 4. Reviewer is right, data are quite old. However, in our opinion, they are still worth to be published. |
| Line 141 | selecting one in every four people amongst (reword) | Revised. |
| Line 147-149 | I struggled to follow this, please reword it | We are not sure to have understood the comment. It is the only way to write the assumption of the sample size calculation |
| Line 155 | was asked whether they resided (add in word) | Revised. |
| Line 172 | estimate the size of the canine population (add in word) | Revised. |
| Line 173 | comma after ‘investigation (9), | Revised. |
| Line 174 | remove as prior | A prior distribution needs to be stated in a Bayesian approach. We put it in italic. |
| Line 174 | comma after registered | Revised. |
| Line 204 | 362,277 dogs were registered in the DR (reword) | Revised. |
| Line 210 | The age distribution of the dog population showed a moderately different shape for gender (reword) | Revised. |
| Line 212 | fed mixed food- delete by | Revised. |
|  | Line 216- dogs are usually referred to as gender rather than sex- please change throughout | Unfortunately, this comment is in contrast to what raised by reviewer#1. We apologize but we can not satisfy both reviewers. |
|  | Table 1 and 2 seem very similar- maybe consider deleting one | Reviewer is right, data of Table 1 are present also in logistic analyses tables, but the way to present it in a simple and direct way (as in Table 1) could be useful to readers that are interested only in demography. They could not find easy to recalculate the characteristics biased by the outcome variable in tables 2 (and 3 and 4). |
| Line 234 | commonly fed commercial or mixed food (reword) | Revised. |
| Line 236 | univariable analysis, while multivariable model (add in comma) | Revised. |
| Line 258 | leishmaniosis- typo- leishmaniasis | Revised. |
| Line 260 | human bite) and the impact (add in word) | Revised. |
| Line 262 | demography of the canine (add in word) | Revised. |
| Line 290 | The percentage of respondents having (make plural) | Revised. |
| Line 292 | what is the human development index? It would be nice to know what this is and what it means | Revised. |
| Line 302 | The wide base of the pyramid (add in word) | Revised. |
| Line 304 | almost identical to that reported worldwide (reword) | Revised. |
| Line 305 | delete hold | Revised. |
| Line 306 | replace ‘resulted greater’ with ‘were more common’ | Revised. |
| Line 310 | reword to ‘register new born puppies, owner and address changes’ | Revised. |
| Line 311 | implementing DRs, while no DRs is (add in comma) | Revised. |
| Line 315 | should be researchers | Revised. |
| Line 326 | in case an animal becomes lost (reword)) | Revised. |
| Line 328/9 | responsibility of an unwanted litter (add in word) | Revised. |
| Line 331 | promoting responsible dog ownership (reword) | Revised. |
| Line 341 | greater than the proportion reported (add in word) | Revised. |
| Line 342 | reword to – ‘and it is worth noting…’ | Revised. |
| Line 350 | order to reduce the stray dog population (add in word) | Revised. |
| Line 360 | similar results, while an Irish National (add in comma) | Revised. |
| Line 369 | commonly performed on an annual basis such as vaccination(add in word) | Revised. |
| Line 376 | abstain from regularly taking the pet- reword | Revised. |
| Line 380 | statistically associated with an annual veterinary visit (add in word) | Revised. |
| Line 381 | Since the cost of veterinary care (add in word) | Revised. |
| Line 388 | usually on a diet with commercial (add in word) | Revised. |
| Line 394/5 | identified as a significant factor by (add in word) | Revised. |
| Line 396 | enough space at their disposal (Add in word) | Revised. |
| Line 397 | we found that the presence of children (add in word) | Revised. |
| Line 407 | Cats were popular in the study area… (reword) | Revised. |
| Line 408/9 | but different to the results of Murray et al (reword) | Revised. |
| Line 410 | replace resulted with were | Revised. |
